# Use of Image-Guided Surgical Navigation during Resection of Locally Recurrent Rectal Cancer

**DOI:** 10.3390/life12050645

**Published:** 2022-04-27

**Authors:** Harald C. Groen, Anne G. den Hartog, Wouter J. Heerink, Koert F. D. Kuhlmann, Niels F. M. Kok, Ruben van Veen, Marijn A. J. Hiep, Petur Snaebjornsson, Brechtje A. Grotenhuis, Geerard L. Beets, Arend G. J. Aalbers, Theo J. M. Ruers

**Affiliations:** 1 Department of Surgical Oncology, Netherlands Cancer Institute, 1066 CX Amsterdam, The Netherlands; a.d.hartog@nki.nl (A.G.d.H.); w.heerink@nki.nl (W.J.H.); k.kuhlmann@nki.nl (K.F.D.K.); n.kok@nki.nl (N.F.M.K.); rvv.vanveen@gmail.com (R.v.V.); ma.hiep@nki.nl (M.A.J.H.); b.grotenhuis@nki.nl (B.A.G.); g.beets@nki.nl (G.L.B.); a.aalbers@nki.nl (A.G.J.A.); t.ruers@nki.nl (T.J.M.R.); 2 Department of Pathology, Netherlands Cancer Institute, 1066 CX Amsterdam, The Netherlands; p.snaebjornsson@nki.nl; 3 Faculty of Science and Technology (TNW), Nanobiophysics Group (NBP), University of Twente, 7500 AE Enschede, The Netherlands

**Keywords:** image-guided surgical navigation, locally recurrent rectal cancer

## Abstract

Surgery for locally recurrent rectal cancer (LRRC) presents several challenges, which is why the percentage of inadequate resections of these tumors is high. In this exploratory study, we evaluate the use of image-guided surgical navigation during resection of LRRC. Patients who were scheduled to undergo surgical resection of LRRC who were deemed by the multidisciplinary team to be at a high risk of inadequate tumor resection were selected to undergo surgical navigation. The risk of inadequate surgery was further determined by the proximity of the tumor to critical anatomical structures. Workflow characteristics of the surgical navigation procedure were evaluated, while the surgical outcome was determined by the status of the resection margin. In total, 20 patients were analyzed. For all procedures, surgical navigation was completed successfully and demonstrated to be accurate, while no complications related to the surgical navigation were discerned. Radical resection was achieved in 14 cases (70%). In five cases (25%), a tumor-positive resection margin (R1) was anticipated during surgery, as extensive radical resection was determined to be compromised. These patients all received intraoperative brachytherapy. In one case (5%), an unexpected R1 resection was performed. Surgical navigation during resection of LRRC is thus safe and feasible and enables accurate surgical guidance.

## 1. Introduction

According to the GLOBOAN cancer incidence database, rectal cancer ranks in eighth position with a cumulative lifetime risk of almost 1% [1]. Surgery is the principal treatment option for rectal cancer, and is often accompanied by neoadjuvant treatment, which consists of radiotherapy, chemotherapy, or a combination of both. Despite improvements in multidisciplinary treatment, the proportion of tumor-positive resection margins after surgical treatment of primary rectal cancer remains 10–15% [2,3]. This, in turn, leads to a local recurrence rate of rectal cancer of 6–10% [4].

The treatment of locally recurrent rectal cancer (LRRC) remains a clinical challenge. Similar to primary rectal cancer, complete resection of LRRC is essential for improving both local control and long-term survival [5]. However, due to prior radiation and surgery, the normal surgical planes are disrupted, thus making it challenging to differentiate between fibrosis and recurrent malignancy. After surgical resection of LRRC, high tumor-positive resection margin (R1) rates are reported of 38–62% [6,7]. In these R1 LRRC cases, the overall five-year survival varies from 10–18% [8,9,10,11,12,13] compared with 48–58% among patients for whom a negative resection margin (R0) is achieved.

To achieve R0 resection in conditions in which someone’s default anatomy has been changed due to prior treatment (radiotherapy or surgery), image-guided surgical navigation might be beneficial. The main goal of surgical navigation is to provide surgeons with an intuitive live-view of where their surgical instruments are positioned in relation to the preoperative images. In addition to improving R0 rate, surgical navigation also has the potential to allow for more precise surgery, insofar as it helps to spare healthy anatomical structures [14]. For some time now, surgical navigation solutions have been commercially available for cranio-maxillofacial, spinal, trauma, orthopedic, and neurosurgery [15,16,17]. However, there is currently no commercial system available for abdominal or pelvic surgery.

A surgical navigation setup for abdominal surgery was developed and evaluated in-house at the Netherlands Cancer Institute (NKI) [18,19]. This setup has resulted in numerous patient studies that demonstrate the added value of the technology [18,19,20,21]. Among patients operated on for LRRC, R0 resection rate using surgical navigation was 79% compared with 49% in a case control group [20].

In the present study, we present how surgical navigation is embedded within our surgical workflow; more specifically, we investigate its use in those cases of LRRC where R0 resection is considered to be extremely challenging due to tumor localization. To this end, we analyzed the use of surgical navigation in a high-risk subpopulation of LRRC. This group consisted of patients in whom intraoperative brachytherapy (IOBT) was considered by the multidisciplinary team (MDT), due to serious doubts over being able to achieve a R0 resection.

## 2. Materials and Methods

### 2.1. Study Design 

For this study, 21 patients with LRRC were selected who were operated on using surgical navigation and scheduled for IOBT in the NKI from March 2018 until December 2021. Up until February 2020, these patients were enrolled in a clinical study (N13NAV/NL43553.031.13), for which patients provided written consent. After February 2020, the surgical navigation setup was available as a standard of care within the NKI. In our hospital, all patients are asked for “permission for further use” of clinically collected data for the purposes of scientific research [22]. Therefore, all the patients presented in this paper provided prior consent. For each patient, after tumor resection, the radiation-oncologist was consulted during surgery whether to apply IOBT or not as part of the standard clinical workflow.

### 2.2. Surgical Navigation

**Figure 1:**
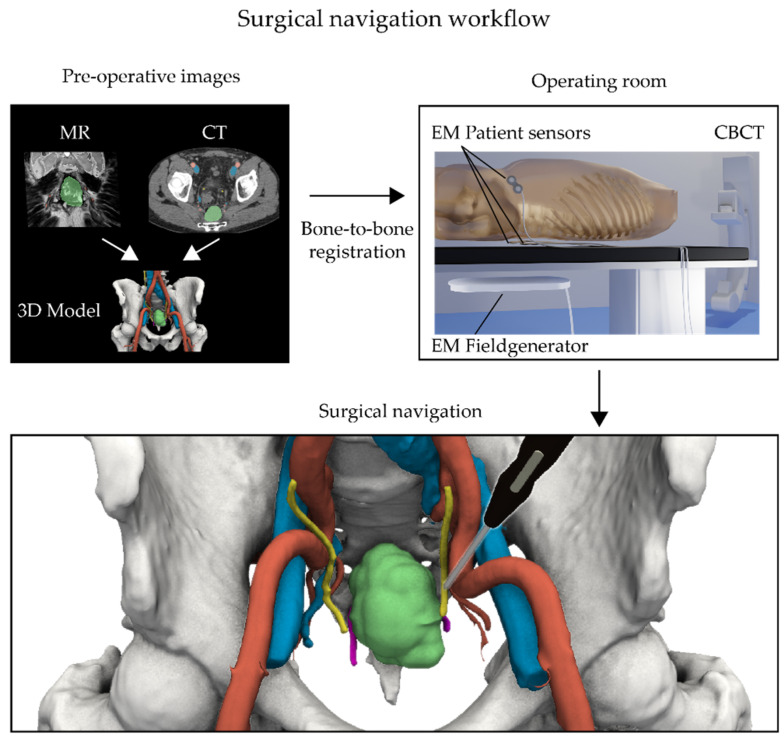
Surgical navigation workflow showing live patient and pointer tracking by an electromagnetic (EM) navigation system. (**Top left**) Prior to surgery, a 3D model of the pelvic area is made by delineating all relevant structures on a preoperative CT and/or MRI scan. (**Top right**) During surgery, the procedure starts by placing EM patient sensors on the skin and imaging the patient’s position on the surgical table using a cone beam CT (CBCT). This image is then subsequently registered to the preoperative CT scan and linked with the 3D model. (**Bottom**) This registration process permits following the movements of the surgeon within the detailed 3D model via the use of an in vivo EM tracked pointer (black pointer).

The surgical navigation technique has been evaluated and described previously by Nijkamp et al. [19]. An overview of both the hardware and different registration steps is presented in Figure 1.

For each patient, the critical anatomical structures were semi-automatically delineated based on standard preoperative clinical images like CT and MRI using in-house developed software (WorldMatch, 8.17b) or 3D Slicer (4.10–4.11, www.slicer.org) [23]. These preoperative images comprised contrast-enhanced CT scans and axial, coronal, and sagittal T2w MRIs, and were in some instances complemented by DWI MRI scans. These segmented structures include the abdominal arteries, veins, bones, and—if visible and required by the surgeon—nerves and ureters. The target tumor(s) were delineated manually based on key images provided by the radiologist and in consultation with the surgeon. All these delineations resulted in a patient-specific color-coded 3D model of the patient (Figure 1, top left). 

To enable surgical navigation in the operating room (OR), this 3D model—and related preoperative images—needs to be registered to the patient’s position on the surgical table. Given that this position can change slightly over the course of the surgery, EM patient sensors (Philips Nederland B.V., Eindhoven, The Netherlands) were taped to the skin as reference points. Ordinarily, two patient sensors were placed on both the left and right side of the lower back at the level of L5 and one at the iliac crest on the front. Depending on the type of surgery and surgical table being used, different combinations of radiolucent bed inserts, EM field generators, and CBCT systems were used in this study (Figure 1, top right).

Tracking of the patient sensors was accomplished using the EM tracking Aurora system (Northern Digital Inc., Waterloo, Ontario, Canada) via either the Tabletop or the Planar field generator. These were mounted below a radiolucent bed insert of the surgical table. Patient registration in the OR was performed by CBCT using the Philips Allura Xper system (Philips Nederland B.V., Eindhoven, The Netherlands) or the Ziehm Vision RFD 3D (Ziehm Imaging, Orlando, FL, USA). For this step, both the patient sensors and the pelvic bones need to be visible inside the acquired CBCT scans. Due to the limited field of view, two separate CBCT scans were acquired for each patient, which were subsequently merged in the registration process. To minimize image artifacts and decrease patient dose, radiolucent bed inserts were used, while the field generator was removed during acquisition if possible (Figure 1, top right).

The in-house developed software SurgNav (v4.20; NKI, Amsterdam, The Netherlands) was used for the surgical navigation procedures. This software package allowed for loading the preoperative images and the 3D models. Subsequently, the CBCT scans were individually registered to the preoperative CT scan using a rigid intensity-based bone-to-bone algorithm, with correlation ratio serving as the similarity measure. Since the positioning of the legs is inherently different from preoperative imaging, a clip-box was used to exclude the femur heads to avoid misalignment. Visual inspection of the registration was performed and, if needed, the registration process was repeated with an altered clip-box. After registration, the position of the patient trackers in the CBCT scan were marked manually on the scan, which, in turn, allowed for real-time registration of the EM coordinates of the patient trackers and the preoperative images (Figure 1, bottom).

The surgeon was provided with a sterile EM tracked 6-degree of freedom (6DOF) Probe (Northern Digital Inc., Waterloo, ON, Canada), whose location was visualized within the registered preoperative images and 3D model. The accuracy of the surgical navigation was determined by placing the probe at anatomical landmarks, such as bifurcations, ureters, sacral promontory, and by visual inspection of the location within the 3D model. If needed, small spatial corrections were made in left–right, cranial–caudal, and anterior–posterior directions with a maximum of 20 mm.

During surgery, the surgeon was free to use the surgical navigation. Depending on the specific surgery, it could be used to, among other things, identify/verify vessels, nerves, and the location/extension of the tumor.

### 2.3. Analysis

SurgNav allows for the constant recording of the EM sensors throughout the surgery, which allows for offline analysis via the use of custom Matlab scripts (R2016a; The MathWorks Inc., Natick, MA, USA). From these data, the duration of the surgical navigation, defined as the length of time that the pointer was used inside the patient, could be extracted.

To quantify the difficulty of the location of the tumors, the shortest distance between the delineated tumor surface and the anatomical structures was determined. In this analysis, only those structures closer than 3 cm were considered relevant and thus taken into account. A “risk” score was calculated based on the inverse shortest distance and normalized to 1. This means that a shortest distance of 3 cm had a low risk (0), while a shortest distance of 0 cm had a high risk (1).

The additional time needed to set up the surgical navigation, that is, attaching the patient sensors and CBCT acquisition, was recorded during the surgery. At the end of each surgery, the surgeon was briefly interviewed and asked to complete a questionnaire pertaining to the use and application of the surgical navigation. The first part of the questionnaire included 10 general questions about the navigation during surgery. The second part included 10 questions (with five answers ranging from “strongly disagree” to “strongly agree”) to determine the system usability score (SUS, range 0–100). In order to have a high chance of acceptance, a SUS of 70 or higher is vital [24]. The third part included six questions (with five answers ranging from “more negative” to “more positive”) drawing comparisons between the conventional setting, that is, surgery without navigation. The answers were used to quantify the effectiveness, efficiency, and decisiveness of the surgical navigation on a 5-point Likert scale. Three points or higher indicates an additional value of surgical navigation technique compared with the conventional surgical technique. To account for an unbalanced evaluation stemming from the number of surgeries carried out by each surgeon, the average scores of each surgeon were used to calculate the overall averaged SUS and Likert score.

Patient information was extracted from the digital patient records, including the final pathology report. A radical resection (R0) was defined as tumor margin > 0 mm. Length of hospital stay was calculated from the day of surgery until the moment of discharge. The total surgery time was calculated from the moment of the first incision up until the closure of the patient, which may include other surgical interventions like the IOBT, urology and/or reconstructive plastic surgery. Postoperative complications were graded according to the Clavien–Dindo classification 30 days after surgery [25].

No statistical tests were performed on the data. If the data were normally distributed, then the mean and standard deviation were reported; otherwise, the median with range (min–max) or interquartile range (IQR) were reported.

## 3. Results

### 3.1. Patient Characteristics

In total, 21 patients were operated on via surgical navigation and scheduled IOBT for LRRC between March 2018 and December 2021. From this group, 15 patients participated in the N13NAV and six were scheduled for navigation as standard clinical care. From the N13NAV group, 10 patients were reported previously in the clinical evaluation of the resection rate compared with a historical cohort [20]. One patient was excluded for further analysis since unexpected severe blood loss and hemodynamic instability changed the course of their surgery substantially, which meant that the navigation was no longer used for tumor resection. 

As such, 20 patients were analyzed, 10 male and 10 female, with a median age of 60 (41–78) years at surgery (see Table 1). All but two patients had previously undergone abdominal surgery. Initially, these two patients had a clinical complete response after neoadjuvant chemoradiotherapy. These patients were closely monitored every 3 to 6 months according to the “watch and wait” policy [26,27,28]; however, during follow-up, a local recurrence was observed. All patients received neoadjuvant treatment prior to surgery with navigation, via either chemotherapy (1), chemoradiation (12), or a combination of both (7). The main tumor location (15) was in the pelvic wall, presacral, or perineal region. Three patients had a staple line recurrence, while two had local recurrence intra- and extra-luminal after the “watch and wait” policy. The type of open surgery ranged from low anterior resection (4), abdominoperineal resection (5), and extensive local resection (5) to a pelvic exenteration (6).

### 3.2. Surgical Navigation

There were no technical failures and the surgical navigation was accurate in all the procedures (see Table 2). The total setup time for the navigation was 16 (9–20) min. Manual fine-tuning of the surgical navigation during surgery based on anatomical structures was required in 17/20 cases, mainly in the caudal–cranial direction with a median of 7 (0–20) mm. In 3/20 cases, the accuracy was checked but no corrections were required. Navigation was available throughout the surgeries, with a median time of 4.2 h (254 min, range 104–553 min). The pointer was used for a median time of 13.7 (3.1–44.7) min, which constitutes 5.6% (1.1–12.5%) of the total time the navigation was available.

**Figure 2:**
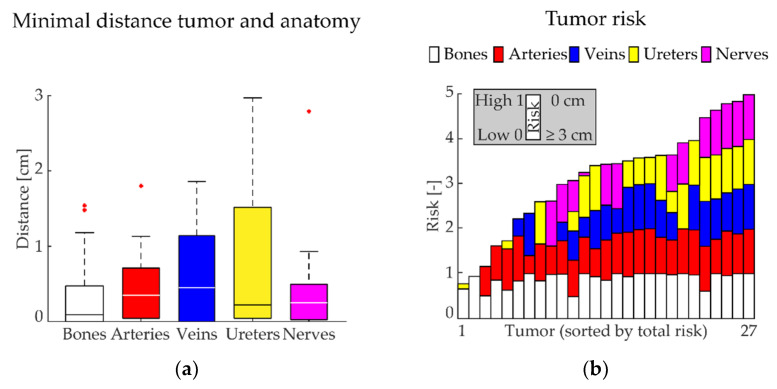
(**a**) For each tumor, the shortest distance of the tumor surface to a critical anatomical structure (bones, arteries, veins, ureters, nerves) was calculated. Values are presented as box plots (median: horizontal line within each bar; the bottom of each bar is defined as the 1st quartile; the top of each bar as the 3rd quartile; outliers are the red points). (**b**) The total risk for each tumor was determined based on this minimal distance. Here, the length of each individual bar is minimal 0 (low risk, which is ≥3 cm between tumor surface and critical anatomical structure) and maximal 1 (high risk, which is a distance of 0 cm between tumor surface and critical anatomical structure). The total risk for each tumor is presented as the sum of all the individual risks.

Overall, 27 tumors were delineated with 1 (1–3) tumor per patient (see Table 3), with a median maximum size of 4.8 (1.5–12.1) cm and a median volume of 10.0 (0.05–125.8) mL. The minimal median (IQR) distances from the tumor surface to the delineated structures were: bones 0.1 (0.5) cm, arteries 0.4 (0.7) cm, veins 0.5 (1.1) cm, ureters 0.2 (1.5) cm, and nerves 0.3 (0.5) cm; see Figure 2a. 

**Figure 3:**
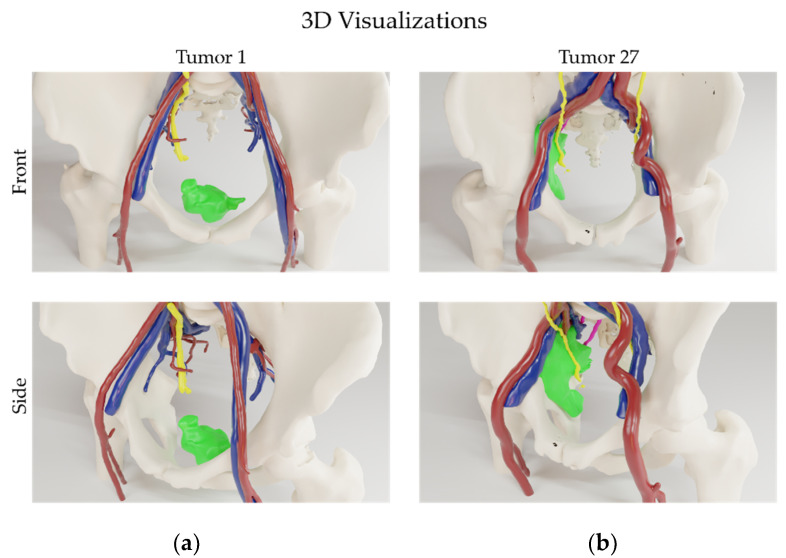
3D visualizations of the two tumors (green) from different patients. (**a**) Tumor 1, only closely related to bone (white) and (**b**) tumor 27, closely related to bone (white), arteries (red), veins (blue), ureters (yellow), and nerves (magenta).

Both the individual risk for each anatomical structure—calculated based on this minimal distance—and the total accumulated risk for each tumor is shown in Figure 2b. The median of the total risk for each tumor was 3.4 (0.8–5.0), see Table 3. Figure 2b shows that all tumors were closely related to one or more critical anatomical structures. For example, the border of tumor 27 was closely related to all the critical anatomical structures analyzed, thus resulting in a high total risk score. Conversely, the border for tumor 1 is only in close relation to bone, thus resulting in a low total risk score. These tumors are visualized in 3D in Figure 3.

### 3.3. Surgical Outcome

The median total duration of the procedures was 7.5 (2.7–14.8) h, with a median blood loss of 2350 (460–9300) mL (see Table 4). IOBT was not used in six surgical procedures, after consultation with the radiation-oncologist during surgery. One relevant intraoperative event—unrelated to the surgical navigation procedure—was observed (bladder damage). Thirteen patients (13/20) showed postoperative complications, none of which were related to the navigation procedure. The median postoperative stay was 8 (3–27) days. 

### 3.4. Pathological Outcome

**Figure 4:**
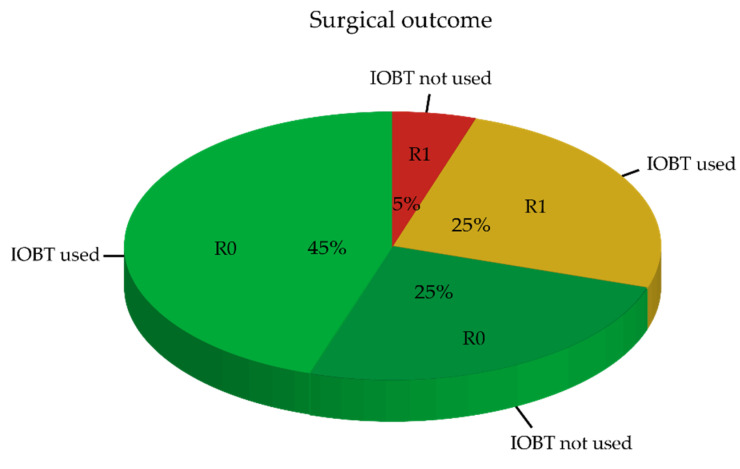
Pathological examination of the excised specimens (R0 = radical, R1 = not radical) in relation to the intraoperative decision to use IOBT. In the event that IOBT was used, an R1 resection was anticipated.

Pathological evaluation of the excised specimens showed an R0 resection rate of 70% (14/20) (Figure 4). Of the 70% radical (R0) resections, 45% (nine patients) received IOBT, while 25% (five patients) did not receive IOBT. Among these five patients, both the surgeon and radiation-oncologist were confident that resection was adequate, and scheduled IOBT was no longer indicated. In the group of six patients (30%) with the non-radical resection (R1), five patients (25%) received IOBT. Among these patients, a more extensive resection was not possible and non-radical resection margins were anticipated by the surgeon and radiation-oncologist, which, in turn, led to the decision to use IOBT. In one of these patients (5%), a positive resection margin was not expected during surgery and thus IOBT was unjustly omitted.

### 3.5. Surgeon Satisfaction

**Figure 5:**
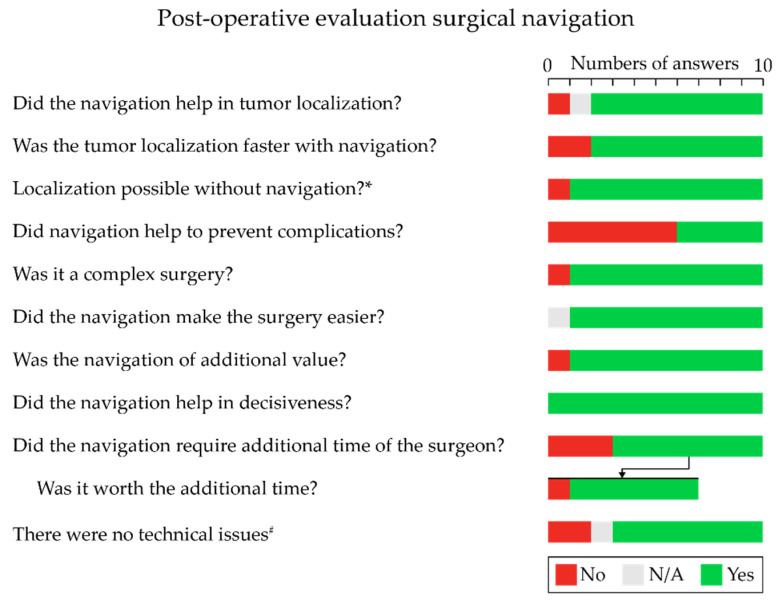
Results of the questionnaire regarding the use and application of surgical navigation. Ten questionnaires completed by seven different surgeons. * yes, but more difficult or lower chance for radical resection (6 cases). ^#^ low accuracy (1 case); accuracy decreased over time (1 case).

Of the 20 navigated procedures, 10 questionnaires were answered by seven different surgeons. The summarized answers to general questions about the navigation during the surgery are presented in Figure 5. In most cases, the surgeon indicated that the surgical procedure was complex, and that the surgical navigation helped to localize the tumor, find it faster, and made the surgery easier. Although the surgery would have been possible without surgical navigation, it was clearly of additional value, insofar as it helped in terms of the decisiveness of the surgery and potentially increased the chance for radical resection. Although surgical navigation did require some additional setup time, it was worthwhile. Some technical issues were reported which were related to a decrease in accuracy during surgery.

**Figure 6:**
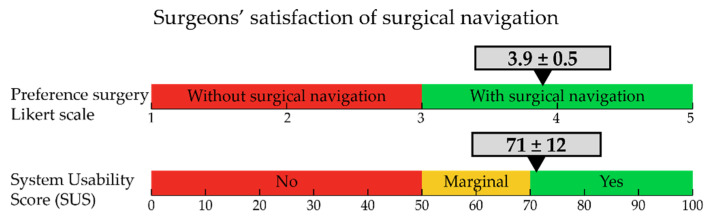
(**Top**) The preference for the innovative surgical navigation expressed in a Likert score (1–5, where >3 is in favor of the new technique). The average value is indicated by the arrow, while the standard deviation is indicated by the grey bar. Values are based on 10 completed questionnaires by seven surgeons. (**Bottom**) Level of surgeon satisfaction expressed in system usability score (SUS, 0–100, where values >70 are considered to be useful).

Figure 6 shows the results of the system usability score and preference score for the navigation technique to be used in the future. The preference to use the navigation technique during surgery was 3.9 ± 0.5 and the average SUS score was 71 ± 12. A score of 70 or higher means that the technology has a high change of acceptance. Although surgeons were positive about the ease of clinical use, they gave low scores to the potential self-support of the technology, thus meaning that technical assistance is still required for the current setup.

## 4. Discussion

The present study evaluated the workflow of surgical navigation among patients undergoing complex surgery for LRRC. The surgical navigation proved to be accurate and supported the surgeons in their decision making during the procedure. The percentage of patients with an unexpected R1 resection was 5%. 

In an exploratory study it remains challenging to determine a homogenous patient population with regard to the complexity of the procedure. We tried to address this issue by selecting only those patients whom the MDT decided that a R0 resection might be difficult to achieve and therefore were considered for IOBT. Despite the expertise of the MDT, it nevertheless still proved to be extremely challenging to correctly predict the actual surgical outcome. Alongside this, we determined the distance of the tumor border to critical anatomical structures. A short distance indicates a high risk for R1 resection, while the more anatomical structures that are close to the tumor border, the higher the total risk. The majority of the tumors were located at a short distance to at least three different critical anatomical structures. This was reflected by a median total risk score of 3.4. This finding is in line with the observation that the surgeons rated the surgeries as complex in their questionnaires. In addition to tumor location and relation to critical anatomical structures, other factors may also have contributed to this judgement, such as, for example, prior surgeries or the effect of preoperative radiotherapy, which adds another level of surgical complexity. 

Correct registration of intraoperative images to preoperative images is needed to facilitate adequate surgical navigation. Overall, the registration accuracy was adequate, although depending on surgical position, minor adjustments were required during the procedure in most cases. Small manual registration corrections based on the anatomy were more prominent if the leg position of the patient in the OR (i.e., supine split-leg position and leg holders) differed from the straight position during the preoperative CT or MR imaging. During the surgical procedure, registration adjustment had to be made when patient sensors moved in relation to the bones, which generally occurred because of a change in the Trendelenburg position. Our results on registration accuracy are comparable to previous results published by our group, showing a registration accuracy of 4 mm for straight setup and 6.3 mm for supine split-leg position [19]. 

The whole navigation procedure turned out to be safe and no complications were noted due to the navigation procedure. Surgeons considered the procedure to be very useful, especially during the decision-making process on determining the resection margins. Unfortunately, only 50% of the postoperative questionnaires were filled out by the surgeons. This was mainly due to the specific time at which these questionnaires were handed to the surgeons; namely after a long day of a complex surgical procedure, which had a detrimental impact upon their level of compliance. 

In this study, we evaluated the technology among a patient population of LRRC, where surgical resection is extremely challenging and results in a notoriously high percentage of inadequate surgical resections (R1). All patients were preoperatively discussed in a multidisciplinary team in which it was decided to schedule the patients for IOBT, as radical resection was expected to be either unlikely or difficult. Despite these challenges, we achieved an R0 resection rate of 70%. In five (25%) patients, a R1 resection was predicted during surgery due to the fact that wider tumor resection was not feasible, and so these patients all underwent IOBT. We observed an unexpected R1 resection in only one patient (5%). Although comparison with extant literature is extremely difficult due to the low number of patients, wide variation between LRRC patients, and selection bias, the results in this study do seem favorable compared with reported R1 resection rates up to 50% in comparable patient populations [6,7]. The outcome of the current study is in line with an earlier report from our group in which surgical navigation for LRRC cancer was compared with a historical control group [20]. Using surgical navigation, an R0 resection rate of 75% was reported, although patient selection might have been more favorable as patients with and without scheduled IOBT were included. 

In our institution, we have thus far used surgical navigation for pelvic surgical procedures in more than 50 patients, in a relatively standardized way. Although improvements in registration procedures and accuracy can still be made, we consider the technology to have reached stage 2b of the IDEAL classification [29,30]. The logical way to proceed and establish clinical value would be to conduct a randomized clinical trial. Unfortunately, the number of procedures for LRRC in a single center are generally too small to run such a trial. In addition, further standardization of the setup is needed. In particular, surgeons should be more self-supporting in using the surgical navigation software, as this would eliminate the current need for technical support. 

## 5. Conclusions

Image-guided surgical navigation is feasible during surgery for locally recurrent rectal cancer. The technology adequately guides surgeons through the surgical procedure by identifying critical structures and the most optimal resection plane. Results on tumor-free resection margins (R0) appear to be favorable in relation to the standard surgical procedure.

## Figures and Tables

**Table 1:** Characteristics of patients operated on for LRRC and scheduled for IOBT.

	Value(s)
Total	20
Sex	10 male, 10 female
Age (years) at the surgery, median (min–max)	60 (41–78)
**Previous surgery**	
No (complete response after “watch and wait”) *	2
Yes	18
**Neoadjuvant treatment for the recurrent**	
Chemotherapy	1
Chemoradiation	12
Chemoradiation + chemotherapy	7
**Tumor location**	
Pelvic wall/presacral/perineal	15
Staple line recurrence	3
Recurrence after “watch and wait” ^#^	2
**Type of surgical resection**	
Open low anterior resection (LAR)	4
Open abdominoperineal resection (APR)	5
Extensive local resection	5
Pelvic exenteration	6

* These patients developed an intra- and extra-luminal recurrence after initial complete response after chemoradiation therapy and follow-up in the “watch and wait” policy. ^#^ Intra- and extra-luminal local recurrence.

**Table 2:** Surgical navigation results; values are presented as either the total number or the median (range min–max).

	Value(s)	
Successful surgical navigations	20 (100%)	
Setup time surgical navigation (min)	16 (9–20)	
**CBCT scanner**		
Allura (hybrid operation room)	15	
Ziehm (mobile 3D C-arm)	5	
**Manual fine-tuning registration during surgery**	Yes	No
Fine-tune required	17	3
Left–Right (mm)	3.0 (0–14)	0
Caudal–Cranial (mm)	7.0 (0–20)	0
Posterior–Anterior (mm)	3.0 (0–13)	0
Surgical position related to fine-tune		
Straight	4	2
Supine split-leg position	7	1
Supine split-leg position, later leg holders	1	-
Leg holders	4	-
Prone	1	-
**Navigation usage**		
Available (min)	254 (104–553)	
Pointer used (min)	13.7 (3.1–44.7)	
Relative pointer used (%)	5.6% (1.1–12.5%)	

**Table 3:** Tumor characteristics; values are presented as either the total number or the median (range min–max).

	Value(s)
Total	27
Tumors per patient	1 (1–3)
Maximum size (cm)	4.8 (1.5–12.1)
Volume (mL)	10.0 (0.5–125.8)
Total risk (0–5)	3.4 (0.8–5.0)

**Table 4:** Surgical outcome; values are presented as either the total number or the median (range min–max).

	Value(s)
Surgical time (h)	7.5 (2.7–14.8)
Blood loss (mL)	2350 (460–9300)
**Intraoperative brachytherapy (IOBT)**	
Not used	6
Used	14
**Unexpected intraoperative events**	
None	19
Bladder damage	1
**Postoperative complications**	
Navigation related	-
Non-navigation related	13
Clavien–Dindo	
I	2
II	2
III	-
IIIa	1
IIIb	7
IV	-
IVa	1
IVb	-
V	-
Hospitalization (days)	8 (3–27)
Pathological evaluation	
Radical (R0)	14
Not radical (R1)	6

## Data Availability

Not applicable.

## References

[1] Sung H., Ferlay J., Siegel R.L., Laversanne M., Soerjomataram I., Jemal A., Bray F. (2021). Global Cancer Statistics 2020: GLOBOCAN Estimates of Incidence and Mortality Worldwide for 36 Cancers in 185 Countries. CA Cancer J. Clin..

[2] Rickles A.S., Dietz D.W., Chang G.J., Wexner S.D., Berho M.E., Remzi F.H., Greene F.L., Fleshman J.W., Abbas M.A., Peters W. (2015). High Rate of Positive Circumferential Resection Margins Following Rectal Cancer Surgery: A Call to Action. Ann. Surg..

[3] Bonjer H.J., Deijen C.L., Abis G.A., Cuesta M.A., van der Pas M.H.G.M., de Lange-de Klerk E.S.M., Lacy A.M., Bemelman W.A., Andersson J., Angenete E. (2015). A Randomized Trial of Laparoscopic versus Open Surgery for Rectal Cancer. N. Engl. J. Med..

[4] Bosman S.J., Holman F.A., Nieuwenhuijzen G.A.P., Martijn H., Creemers G.J., Rutten H.J.T. (2014). Feasibility of reirradiation in the treatment of locally recurrent rectal cancer. Br. J. Surg..

[5] Cyr D.P., Zih F.S., Wells B.J., Swett-Cosentino J., Burkes R.L., Brierley J.D., Cummings B., Smith A.J., Swallow C.J. (2020). Long-term outcomes following salvage surgery for locally recurrent rectal cancer: A 15-year follow-up study. Eur. J. Surg. Oncol..

[6] Nielsen M., Rasmussen P., Pedersen B., Hagemann-Madsen R., Lindegaard J., Laurberg S. (2015). Early and Late Outcomes of Surgery for Locally Recurrent Rectal Cancer: A Prospective 10-Year Study in the Total Mesorectal Excision Era. Ann. Surg. Oncol..

[7] Gao Z., Gu J. (2021). Surgical treatment of locally recurrent rectal cancer: A narrative review. Ann. Transl. Med..

[8] Kusters M., Dresen R.C., Martijn H., Nieuwenhuijzen G.A., van de Velde C.J.H., van den Berg H.A., Beets-Tan R.G.H., Rutten H.J.T. (2009). Radicality of resection and survival after multimodality treatment is influenced by subsite of locally recurrent rectal cancer. Int. J. Radiat. Oncol. Biol. Phys..

[9] Dresen R.C., Gosens M.J., Martijn H., Nieuwenhuijzen G.A., Creemers G.J., Daniels-Gooszen A.W., van den Brule A.J., van den Berg H.A., Rutten H.J. (2008). Radical resection after IORT-containing multimodality treatment is the most important determinant for outcome in patients treated for locally recurrent rectal cancer. Ann. Surg. Oncol..

[10] Alberda W.J., Verhoef C., Schipper M.E.I., Nuyttens J.J., Rothbarth J., De Wilt J.H.W., Burger J.W.A. (2015). The Importance of a Minimal Tumor-Free Resection Margin in Locally Recurrent Rectal Cancer. Dis. Colon Rectum.

[11] Hagemans J.A.W., van Rees J.M., Alberda W.J., Rothbarth J., Nuyttens J.J.M.E., van Meerten E., Verhoef C., Burger J.W.A. (2020). Locally recurrent rectal cancer; long-term outcome of curative surgical and non-surgical treatment of 447 consecutive patients in a tertiary referral centre. Eur. J. Surg. Oncol..

[12] Mannaerts G.H., Martijn H., Rutten H.J., Hanssens P.E., Wiggers T. (2001). [Local tumor control and (disease-free) survival after surgery with pre- and intraoperative radiotherapy for primary non-resectable rectal carcinoma and local recurrence]. Ned. Tijdschr. Geneeskd..

[13] Holman F.A., Bosman S.J., Haddock M.G., Gunderson L.L., Kusters M., Nieuwenhuijzen G.A.P., van den Berg H., Nelson H., Rutten H.J. (2017). Results of a pooled analysis of IOERT containing multimodality treatment for locally recurrent rectal cancer: Results of 565 patients of two major treatment centres. Eur. J. Surg. Oncol..

[14] Shuman W.H., Valliani A.A., Chapman E.K., Martini M.L., Neifert S.N., Baron R.B., Schupper A.J., Steinberger J.M., Caridi J.M. (2022). Intraoperative Navigation in Spine Surgery: Effects on Complications and Reoperations. World Neurosurg..

[15] Mongen M.A., Willems P.W.A. (2019). Current accuracy of surface matching compared to adhesive markers in patient-to-image registration. Acta Neurochir..

[16] Elfring R., De La Fuente M., Radermacher K. (2010). Assessment of optical localizer accuracy for computer aided surgery systems. Comput. Aided Surg..

[17] Gumprecht H.K., Widenka D.C., Lumenta C.B. (1999). BrainLab VectorVision Neuronavigation System: Technology and clinical experiences in 131 cases. Neurosurgery.

[18] Nijkamp J., Kuhlmann K., Sonke J.-J., Ruers T. (2016). Image-guided navigation surgery for pelvic malignancies using electromagnetic tracking. Proceedings of the Medical Imaging 2016: Image-Guided Procedures, Robotic Interventions, and Modeling.

[19] Nijkamp J., Kuhlmann K.F.D., Ivashchenko O., Pouw B., Hoetjes N., Lindenberg M.A., Aalbers A.G.J., Beets G.L., van Coevorden F., Kok N. (2019). Prospective study on image-guided navigation surgery for pelvic malignancies. J. Surg. Oncol..

[20] Kok E.N.D., van Veen R., Groen H.C., Heerink W.J., Hoetjes N.J., van Werkhoven E., Beets G.L., Aalbers A.G.J., Kuhlmann K.F.D., Nijkamp J. (2020). Association of Image-Guided Navigation With Complete Resection Rate in Patients With Locally Advanced Primary and Recurrent Rectal Cancer. JAMA Netw. Open.

[21] Reijers S.J.M., Heerink W.J., van Veen R., Nijkamp J., Hoetjes N.J., Schrage Y., van Akkooi A., Beets G.L., van Coevorden F., Ruers T.J.M. (2021). Surgical navigation for challenging recurrent or pretreated intra-abdominal and pelvic soft tissue sarcomas. J. Surg. Oncol..

[22] van Leeuwenhoek A. Consent. https://www.avl.nl/ons-onderzoek-het-nederlands-kankerinstituut/consent/.

[23] Fedorov A., Beichel R., Kalpathy-Cramer J., Finet J., Fillion-Robin J.C., Pujol S., Bauer C., Jennings D., Fennessy F., Sonka M. (2012). 3D Slicer as an image computing platform for the Quantitative Imaging Network. Magn. Reson. Imaging.

[24] Bangor A., Kortum P.T., Miller J.T. (2008). An Empirical Evaluation of the System Usability Scale. Intl. J. Hum. –Comput. Interact..

[25] Dindo D., Demartines N., Clavien P.A. (2004). Classification of surgical complications: A new proposal with evaluation in a cohort of 6336 patients and results of a survey. Ann. Surg..

[26] Maas M., Beets-Tan R.G.H., Lambregts D.M.J., Lammering G., Nelemans P.J., Engelen S.M.E., van Dam R.M., Jansen R.L.H., Sosef M., Leijtens J.W.A. (2011). Wait-and-see policy for clinical complete responders after chemoradiation for rectal cancer. J. Clin. Oncol..

[27] Martens M.H., Maas M., Heijnen L.A., Lambregts D.M.J., Leijtens J.W.A., Stassen L.P.S., Breukink S.O., Hoff C., Belgers E.J., Melenhorst J. (2016). Long-term Outcome of an Organ Preservation Program After Neoadjuvant Treatment for Rectal Cancer. JNCI J. Natl. Cancer Inst..

[28] van der Valk M.J.M., Hilling D.E., Bastiaannet E., Meershoek-Klein Kranenbarg E., Beets G.L., Figueiredo N.L., Habr-Gama A., Perez R.O., Renehan A.G., van de Velde C.J.H. (2018). Long-term outcomes of clinical complete responders after neoadjuvant treatment for rectal cancer in the International Watch & Wait Database (IWWD): An international multicentre registry study. Lancet.

[29] McCulloch P., Altman D.G., Campbell W.B., Flum D.R., Glasziou P., Marshall J.C., Nicholl J. (2009). No surgical innovation without evaluation: The IDEAL recommendations. Lancet.

[30] Hirst A., Philippou Y., Blazeby J., Campbell B., Campbell M., Feinberg J., Rovers M., Blencowe N., Pennell C., Quinn T. (2019). No Surgical Innovation Without Evaluation: Evolution and Further Development of the IDEAL Framework and Recommendations. Ann. Surg..

