# Correction: Groen et al. Use of Image-Guided Surgical Navigation during Resection of Locally Recurrent Rectal Cancer. *Life* 2022, *12*, 645

**DOI:** 10.3390/life13030678

**Published:** 2023-03-02

**Authors:** Harald C. Groen, Anne G. den Hartog, Wouter J. Heerink, Koert F. D. Kuhlmann, Niels F. M. Kok, Ruben van Veen, Marijn A. J. Hiep, Petur Snaebjornsson, Brechtje A. Grotenhuis, Geerard L. Beets, Arend G. J. Aalbers, Theo J. M. Ruers

**Affiliations:** 1 Department of Surgical Oncology, Netherlands Cancer Institute, 1066 CX Amsterdam, The Netherlands; 2 Department of Pathology, Netherlands Cancer Institute, 1066 CX Amsterdam, The Netherlands; 3 Faculty of Science and Technology (TNW), Nanobiophysics Group (NBP), University of Twente, 7500 AE Enschede, The Netherlands

The authors wish to make the following corrections to this paper [1].

In the published version, the resection rate numbers as reported by Kok et al. [2] were cited incorrectly in the Introduction section on page 2.

The sentence “Among patients operated on for LRRC, R0 resection rate using surgical navigation was 21% compared with 51% in a case control group” should be changed to “Among patients operated on for LRRC, R0 resection rate using surgical navigation was 79% compared with 49% in a case control group”.

The authors state that the scientific conclusions are unaffected. This correction was approved by the Academic Editor. The original publication has also been updated.

## References

[1] Groen H.C., den Hartog A.G., Heerink W.J., Kuhlmann K.F.D., Kok N.F.M., van Veen R., Hiep M.A.J., Snaebjornsson P., Grotenhuis B.A., Beets G.L. (2022). Use of Image-Guided Surgical Navigation during Resection of Locally Recurrent Rectal Cancer. Life.

[2] Kok E.N.D., van Veen R., Groen H.C., Heerink W.J., Hoetjes N.J., van Werkhoven E., Beets G.L., Aalbers A.G.J., Kuhlmann K.F.D., Nijkamp J. (2020). Association of Image-Guided Navigation with Complete Resection Rate in Patients with Locally Advanced Primary and Recurrent Rectal Cancer. JAMA Netw. Open.

